# Hyperspectral Imaging during Normothermic Machine Perfusion—A Functional Classification of Ex Vivo Kidneys Based on Convolutional Neural Networks

**DOI:** 10.3390/biomedicines10020397

**Published:** 2022-02-07

**Authors:** Florian Sommer, Bingrui Sun, Julian Fischer, Miriam Goldammer, Christine Thiele, Hagen Malberg, Wenke Markgraf

**Affiliations:** Institute of Biomedical Engineering, Technische Universität Dresden, 01307 Dresden, Germany; florian.sommer@tu-dresden.de (F.S.); bingrui.sun@tu-dresden.de (B.S.); julian.fischer@tu-dresden.de (J.F.); miriam.goldammer@tu-dresden.de (M.G.); christine.thiele@tu-dresden.de (C.T.); hagen.malberg@tu-dresden.de (H.M.)

**Keywords:** normothermic machine perfusion, organ preservation, kidney, biomedical optical imaging, hyperspectral imaging, machine learning, convolutional neural network, residual neural network, classification, function assessment

## Abstract

Facing an ongoing organ shortage in transplant medicine, strategies to increase the use of organs from marginal donors by objective organ assessment are being fostered. In this context, normothermic machine perfusion provides a platform for ex vivo organ evaluation during preservation. Consequently, analytical tools are emerging to determine organ quality. In this study, hyperspectral imaging (HSI) in the wavelength range of 550–995 nm was applied. Classification of 26 kidneys based on HSI was established using KidneyResNet, a convolutional neural network (CNN) based on the ResNet-18 architecture, to predict inulin clearance behavior. HSI preprocessing steps were implemented, including automated region of interest (ROI) selection, before executing the KidneyResNet algorithm. Training parameters and augmentation methods were investigated concerning their influence on the prediction. When classifying individual ROIs, the optimized KidneyResNet model achieved 84% and 62% accuracy in the validation and test set, respectively. With a majority decision on all ROIs of a kidney, the accuracy increased to 96% (validation set) and 100% (test set). These results demonstrate the feasibility of HSI in combination with KidneyResNet for non-invasive prediction of ex vivo kidney function. This knowledge of preoperative renal quality may support the organ acceptance decision.

## 1. Introduction

Terminal renal insufficiency is characterized by irreversible deterioration of kidney function. The only therapeutic option at this stage of the disease is renal replacement. Temporarily, this is possible with the help of dialysis procedures, but only a kidney transplant offers long-term improvement in quality of life and life expectancy [1].

However, the persistent organ shortage has limited this curative treatment procedure since the beginning of transplantation medicine. Currently, the average waiting time for a kidney transplant in the Eurotransplant region is four years [2]. Several approaches have been proposed in the past to minimize the discrepancy between organ availability and demand. These include measures to improve the use of suboptimal donor organs by establishing appropriate criteria for accepting organs for transplantation [3,4,5]. However, the evaluation criteria proposed to date, based e.g., on histological markers or readily available donor characteristics, have limited predictive power for postoperative graft function [6,7].

Preservation of organs by machine perfusion instead of clinically established static cold storage offers new possibilities for organ evaluation in transplantation medicine. Normothermic machine perfusion (NMP), which simulates near-physiological conditions by delivering essential nutrients and oxygen to the organ at endogenous temperatures, is particularly promising for this purpose [8]. With this preservation technique, the metabolism and function of the organ can be maintained ex vivo [8]. Therefore, NMP provides the ability to derive graft function or damage during preservation by examining perfusate, urine, or tissue markers, as well as hemodynamic characteristics [9,10,11].

However, after nearly two decades of intensive research in NMP, little attention has been paid to developing strategies to evaluate renal function. Preliminary promising results show that markers such as lactate clearance, acid-base homeostasis, oxygen consumption, neutrophil gelatin-associated lipocalin, endothelin-1, nanoparticles, flavin mononucleotide, and intrarenal resistance may be suitable to predict post-operative organ function [12,13,14,15]. Furthermore, a quality assessment score (QAS) based on renal blood flow, total urine output, and the subjectively assessable parameter of macroscopic appearance has been proposed to evaluate the quality of human kidneys that were rejected for transplantation [15,16,17]. In the above studies, markers are measured during NMP and their predictive value is assessed after transplantation. In contrast, our recently published study on renal preservation suggests new possibilities for organ evaluation with respect to inulin clearance, which is the gold standard for determining glomerular filtration rate (GFR) in vivo and thus renal function [18]. Knowledge about the ability of the kidneys to filter inulin during NMP allows assessment of functional renal status ex vivo and, if postoperative renal function is also confirmed, may be a promising approach for the development of new preoperative strategies to evaluate renal function [18]. For the first time, perfusion characteristics during NMP (renal blood flow, mean arterial pressure, intrarenal resistance, total urine output) could be correlated with renal function before transplantation [18]. The consequent next step is to expand the assessment strategy and investigate other markers or analytical methods that would provide a more comprehensive measure of organ quality and thus ensure an objective, systematic classification of kidneys according to their function.

A new approach for detecting physiological and pathological properties in tissues and organs is the application of hyperspectral imaging (HSI) [19]. By combining imaging and spectroscopy, local information on morphological features and spectral information on the chemical composition of the tissue can be obtained non-invasively [19]. HSI has demonstrated its potential in numerous medical applications, such as tumor detection [20,21,22], skin analysis [23], and hemorrhagic shock analysis [24]. In addition, various tissue parameters have been calculated from HSI data, such as tissue oxygen saturation [25,26] and tissue water content [27,28]. Knowledge about tissue-specific properties may provide additional information regarding organ quality in transplantation medicine. HSI systems can accurately measure the visible and near-infrared (VIS/NIR)-spectroscopic chemical properties of biological objects such as organ transplants or blood [29]. Consequently, HSI is used for image-guided surgery in organ transplantation and organ preservation [25,27,29,30,31,32,33]. In renal transplantation, there is preliminary evidence of a possible relationship between renal tissue parameters measured with HSI and postoperative GFR [30]. HSI in organ preservation was first applied during NMP of kidneys [31,32]. Further research in this field has identified changes in tissue oxygen saturation, tissue water content, and ischemia-reperfusion injury (IRI) in ex vivo kidneys [25,27,31,32,33]. Recently, HSI has also been used to monitor normothermically perfused livers [34].

To extract tissue-specific features from HSI data, machine learning (ML) techniques are widely used [35]. ML algorithms such as convolutional neural networks (CNNs) and support vector machines (SVMs) have proven to be reliable tools in distinguishing healthy from damaged tissue [35]. CNNs, which have become state of the art for image classification, are increasingly used to analyze HSI data [36]. Previously, studies have primarily focused on the classification of histological tissue types (including breast [37], gastric [38], head and neck [39] cancers) and in vivo real-time tumor segmentation in neurosurgery [40]. To the best of our knowledge, no research has been published regarding the assessment of ex vivo kidneys using HSI and CNNs.

The aim of this work is the functional classification of normothermic perfused kidneys based on HSI data using CNNs, specifically by means of residual neural network (ResNet) architecture. For this purpose, the acquisition of HSI data in a physiological ex vivo environment, its preprocessing, and subsequent classification with CNNs were performed. Using different training parameters and data augmentation methods, various CNN models were trained based on absorbance and reflectance data. Inulin clearance served as a reference for the functional status of the kidneys.

Judging by our promising results, the combination of HSI and ML could become an essential tool for transplanters to assess the condition of an organ, thereby facilitating the decision to accept an organ for transplantation.

## 2. Materials and Methods

### 2.1. Study Design

This exploratory analysis aimed to perform a functional classification of ex vivo kidneys by CNN models based on tissue-related properties obtained by HSI. For this purpose, HSI data recorded during a previously published study [18] were analyzed. Kidneys from healthy laboratory pigs were removed and perfused ex vivo with NMP for four hours. The renal function was assessed by inulin clearance. Hyperspectral images were acquired at specific time points during NMP. To enable an automated function-related evaluation of organs, the HSI data were analyzed using CNNs.

### 2.2. Normothermic Machine Perfusion

The NMP method including the description of blood and organ retrieval, the ex vivo normothermic machine perfusion device, the experimental protocol, and the inulin clearance analysis has been described in detail previously [18]. In brief, kidneys (n = 28) were collected from laboratory pigs of different races, sexes, and body weights after nonabdominal surgical training. After exposition to different periods of warm ischemia time (WIT), the kidneys were stored on ice. Afterwards, the organs were preserved with autologous whole blood for four hours under nearly physiological conditions. Perfusate samples were collected for the determination of inulin clearance. Additionally, hyperspectral images of the kidneys were acquired before NMP and at regular 20-min intervals during renal preservation.

### 2.3. Hyperspectral Imaging System

A detailed description of the HSI system used in this study has already been published [27,29]. The HSI acquisition system comprised two main components: the HSI camera and the illumination unit. The HSI camera operated with the pushbroom scanning method (TIVITA Tissue Camera, Diaspective Vision GmbH, Am Salzhaff, Germany). It provides a 1280 px x 960 px spatial resolution and a spectral resolution of 5 nm covering the wavelength range of 500 nm–995 nm. The distance between the objective and kidney surface was adjusted to 46 cm. For homogeneous illumination of the field of view, the illumination unit consisted of six 20 W quartz-tungsten-halogen spots (OSRAM 41861 Decostar 51 ALU, Osram GmbH, Munich, Germany) with aluminum reflectors. The software TIVITA Suite 0.6.1.4 provided by the manufacturer of the HSI camera was used exclusively for recording the HSI data.

MATLAB (MATLAB R2018b, The MathWorks, Inc., Natick, MA, USA) scripts were implemented to preprocess the HSI data. The following classification of the HSI data using CNNs was implemented in Python (Python 3.8.8, Python Software Foundation, Wilmington, DE, USA).

### 2.4. HSI Data Acquisition and Data Correction

Due to slight variations in illumination conditions during data acquisition, a correction of the raw intensity image is required first. Therefore, for each raw intensity image of a kidney I_RAWx,y,λ_, a dark current image I_DARKx,y,λ_ and a white reference image I_WHITEx,y,λ_ were acquired.

I_WHITEx,y,λ_ was obtained using a white surface board with a uniform and high reflectance (Zenith Polymer Target SG3210, SphereOptics GmbH, Herrsching, Germany). I_DARKx,y,λ_ was acquired while the camera lens was completely covered with its opaque cap. From the raw intensity image I_RAWx,y,λ_, both the reflectance image I_REFLx,y,λ_ and the absorbance image I_ABSx,y,λ_ were calculated (see Equations (1) and (2), [19]):

(1) $$I_{\mathrm{REFL}\;x,y,\lambda }=\frac{I_{\mathrm{RAW}\;x,y,\lambda }-{\;I}_{\mathrm{DARK}\;x,y,\lambda }}{I_{\mathrm{WHITE}\;x,y,\lambda }-{\;I}_{\mathrm{DARK}\;x,y,\lambda }},$$

(2) $$I_{\mathrm{ABS}\;x,y,\lambda }=-\mathrm{lg}I_{\mathrm{REFL}\;x,y,\lambda }.$$

### 2.5. HSI Data Preprocessing

Before the HSI images could be used as input data for the CNN, several image preprocessing steps were required to remove irrelevant information and noise from the data as well as to extract kidney regions to be fed into the CNN. Preprocessing was performed for each acquisition time point of a HSI data cube and included the following steps:

(1) Manual background segmentation,
(2) Wavelength range selection (550 nm–995 nm),
(3) Automated region of interest (ROI) selection,
(4) Vector normalization,
(5) Savitzky-Golay smoothing.

**Figure 1:**
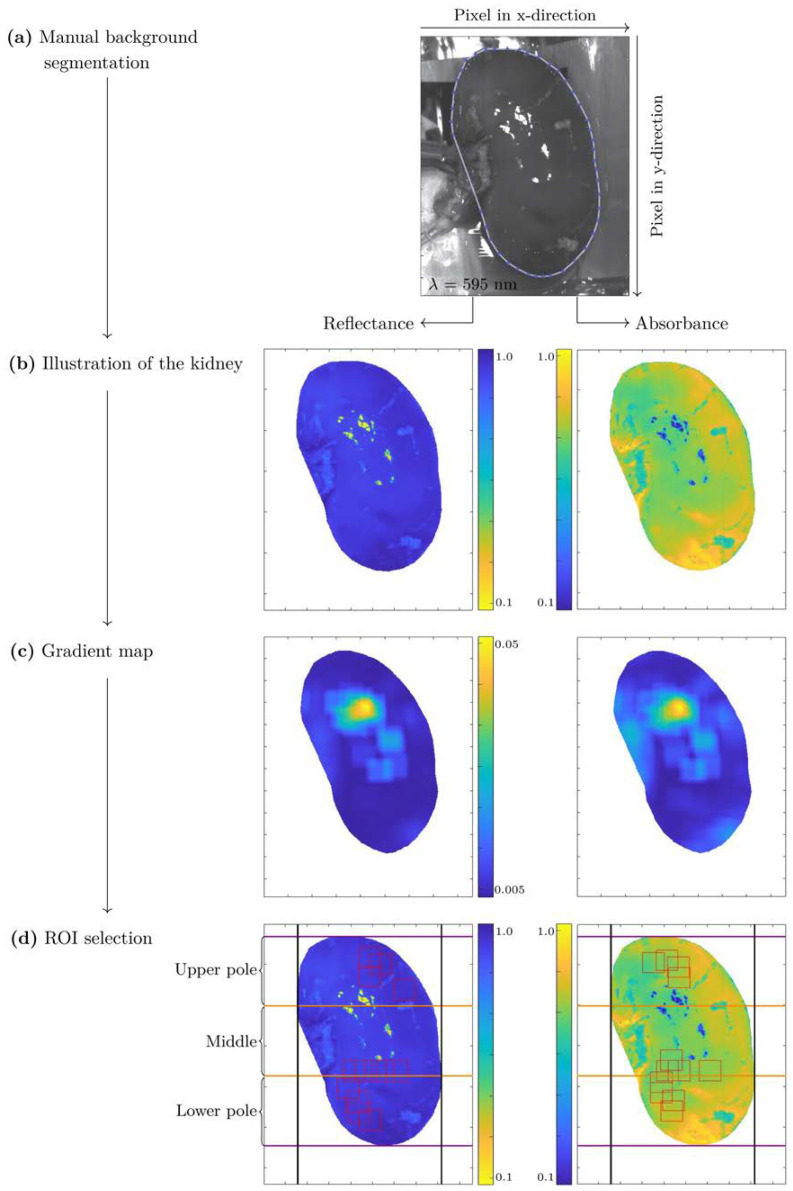
Procedure of region of interest (ROI) selection. The blue and white lines mark the outer boundaries of the kidney (**a**). After segmentation of the background (**b**), calculation of the gradient map (**c**) is performed for the absorbance and reflectance image. The most homogeneous areas are selected as ROI (shown as red squares) (**d**). The orange lines separate the kidney poles from each other.

Automated ROI selection (3) was implemented specifically for this analysis, while the corresponding description of the methods (1), (2), (4), and (5) can be found in [27]. After manual background segmentation (see Figure 1a,b) and wavelength range selection, the HSI images of the kidneys were divided equally into three physiological regions—upper pole, middle, and lower pole. The ROI selection algorithm aims to detect four ROIs with a size of 50 px × 50 px in each physiological region. The criteria for the selection of the ROIs were, that the ROIs have as homogeneous intensity values as possible at the isosbestic point of λ = 805 nm, and that the ROI areas should not overlap by more than 50% to ensure pixel diversity amongst extracted ROIs.

For this purpose, a grid of potential ROIs was rastered on the segmented organ. The corresponding algorithm for calculating homogeneity within each potential ROI (H_ROI_) first calculated the Euclidean distance d between neighboring points in x- and y-direction of the image, respectively. This is demonstrated in Equation (3) for the x-direction with index i corresponding to the positions of a pixel in the ROI in the x-direction and index j accordingly corresponding to the y-direction. Thus, p_i,j_ is one pixel’s intensity, and p_i+1,j_ is the neighboring pixel’s intensity (see Equation (3)).

(3) $$d_{i}\left(j\right)=\sqrt{{\left(p_{i,j}-p_{i+1,j}\right)}^{2}}$$

Second, H_ROI_ of each potential ROI is determined by calculating the average of all Euclidean distances in the x- and y-directions within the ROI (see Equation (4)). Here, k corresponds to the size of the ROI in the x-direction and l corresponds to the size of the ROI in the y-direction.

(4) $$H_{\mathrm{ROI}}=\frac{1}{k\cdot l}\;\left(\overset{k-1}{\underset{i=1}{\sum }}\overset{l}{\underset{j=1}{\sum }}d_{i}+\overset{k}{\underset{i=1}{\sum }}\overset{l-1}{\underset{j=1}{\sum }}d_{j}\right)$$

A small value H_ROI_ corresponds to a high homogeneity in the ROI. The results of each H_ROI_ were merged and plotted on a gradient map, thereby the value of H_ROI_ was assigned to the gradient map pixel in the upper left corner of the ROI (see Figure 1c). H_ROI_ was only calculated if all pixels of the ROI were within the segmented kidney and thus no pixel originated from the background. Four ROIs were selected in each of the three physiological regions (upper pole, middle, and lower pole) by smallest H_ROI_. The overlap to another ROI or physiological region had to be less than 50%. The extracted ROIs represent the minima of the gradient map (see Figure 1c,d), that exhibited the highest homogeneity within the data and contained no specular reflections.

### 2.6. Data Set

The data set included the HSI data of all kidneys that were normothermically perfused during our recently published study [18]. From the 28 kidneys preserved, complete HSI data were acquired from 26 kidneys. Kidneys (n = 2) were not included in the study if perfusion had to be interrupted before 240 min had elapsed.

The data of one kidney consisted of 14 HSI images acquired at different time points. For each acquisition time point, 12 ROIs were generated. In total, 168 ROIs from each kidney were available to analyze tissue-specific features depending on the kidney function. The kidneys of laboratory pigs were assigned to three classes, based on the inulin excretion behavior derived from the GFR and the percentage of inulin eliminated from the blood during NMP (I_e,total_) [18]:

Class 1: nonfunctional kidneys,

Class 2: limited functional kidneys,

Class 3: functional kidneys.

HSI data were labeled according to the functional status of each kidney, thus all 168 ROIs have the same label. A stratified train-test split was performed, resulting in a grouping of *n* = 20 kidneys for the training and validation data set and *n* = 6 kidneys for the test data set (*n* = 6). The properties of all kidneys in the respective functional classes, the kidneys in the test, and training and validation data set are listed in Table 1.

### 2.7. Normalized Cross Correlation

Since kidneys from pigs of different races, sexes, and body weights were the basis for the test data set and training and validation data set, the data were analyzed with regard to its suitability for functional classification of kidneys. Therefore, special attention was paid to tissue-specific characteristics that depend on the quality of the kidneys and not on the characteristics of the pigs. For this reason, before CNN modeling, spectra from bloodless kidneys (*n* = 6) were compared by normalized cross-correlation using the MATLAB function *xcorr*.

Pearson’s correlation coefficient r was used as a measure of linear correlation (see Equation (5)).

(5) $$r=\frac{\sum_{i=1}^{n}\left(x_{i}-\bar{x}\right)\left(y_{i}-\bar{y}\right)}{\sqrt{\sum_{i=1}^{n}{\left(x_{i}-\bar{x}\right)}^{2}\cdot \sum_{i=1}^{n}{\left(y_{i}-\bar{y}\right)}^{2}}}$$

where x_i_ corresponds to the wavelength values (in nm), $\bar{x}$ corresponds to the mean value of the wavelength, y_i_ corresponds to the absorbance values (in a.u.), and $\bar{y}$ corresponds to the mean value of the absorbance. Pearson’s correlation coefficient r is a frequently used parameter to establish a relationship between two variables and is also suitable for comparing spectra (e.g., IR spectra [41]). It takes values between −1 and 1 and were interpreted as follows: 0 to 0.3 (0 to −0.3), negligible; 0.3 to 0.5 (−0.3 to −0.5), low; 0.5 to 0.7 (−0.5 to −0.7), moderate; 0.7 to 0.9 (−0.7 to −0.9), high; and 0.9 to 1.0 (−0.9 to −1.0), very high positive (negative) correlation [42].

### 2.8. CNN Model Architecture and Optimization

In the present work, the Residual Neural Network 18 (ResNet-18) [43], which is a special type of a CNN network architecture, was used. The ResNet-18 implemented will be referred to as KidneyResNet in the following. The KidneyResNet is composed of 18 layers, 16 of which are hidden layers. These 16 layers are structured in four Residual Blocks, each followed by a pooling layer. The number of connecting channels within the residual blocks systematically increases: The two layers in the first residual block have 64 channels. The number of channels doubles with each residual block, resulting in 512 channels in the fourth block. The final layer is a fully connected layer, which combines the extracted features and performs the final classification. See the model summary with additional comments in Table S1 for more detailed information. The implementation of the KidneyResNet was realized in the development environment PyTorch [44].

In a first model optimization phase, some data and training parameters were examined. Using the early stopping method, the optimal number of epochs was identified from the median of the model epochs during validation. Furthermore, the influence of the data origin was analyzed, and three training parameters—dropout, adaptive weights, and learning rate—were investigated. The corresponding variants of the parameters are shown in Table 2.

Using the thereby optimized KidneyResNet model, the data set was enlarged by data augmentation. Table 3 lists the methods studied in this work.

### 2.9. Validation Strategy

The distribution of the data set into training and validation (75%) and test (25%) data set was performed stratified but otherwise randomly. For optimization purposes, the training and validation data set was once more split into the training data set on which the KidneyResNet model was trained and a validation data set to evaluate the generalization ability of the model. Due to the overall small data set, the performance of the KidneyResNet was evaluated using the leave-one-out cross-validation. To be precise, each single kidney was used as validation data set once, with all its 168 ROIs. This kidney-wise split prevented the model from learning specific characteristics of a single kidney during the training process.

For the actual training process, the training data set was fed into the KidneyResNet during the training process while withholding the validation data until the end of each training epoch. The error from the training data set was backpropagated applying the Adam optimizer [45].

However, leave-one-out cross-validation was only applied during the optimization process. For our final model, we used optimized parameters to retrain the model using all training and validation data without a validation split. This final model was applied to the data of the hold-out test data set.

### 2.10. KidneyResNet Model Evaluation

The basis for evaluating the classification quality was the confusion matrix, in which the predicted class was plotted against the actual class. The evaluation criteria accuracy, precision, and recall were derived from the confusion matrix.

Accuracy is the ratio of the sum of all kidneys classified as true positives and true negatives to the total number of classified kidneys. Precision corresponds to the quotient of kidneys classified as true positives across all classes and the total number of kidneys classified as true positives and false positives across all classes. Recall describes the ratio of kidneys classified as true positives across all classes to the total number of kidneys classified as true positives and false negatives across all classes.

During the leave-one-out cross-validation, metrics were calculated as mean results of all validation folds.

## 3. Results

### 3.1. Spectra Comparison of the Kidneys According to the Pig Characteristics

The basis of this study is an inhomogeneous data set that includes kidneys from pigs with different characteristics. To verify the suitability of the data set for the classification task, spectra from bloodless kidneys were compared as a function of race, sex, body weight, and ischemia time using normalized cross-correlation (see Table 4). Two WIT ranges were selected for the study, so that WIT periods were physiologically congruent. This was necessary because possible IRI-induced tissue damage at WIT ≥ 25 min could affect spectral tissue properties [18,33]. Therefore, only groups with corresponding WIT (see Table 4, 1–3 and 4–6) were compared.

The kidney spectra showed very high positive agreement with r = 0.991–0.998 at low and high WIT (see Table 5). Therefore, it was assumed that the kidneys of different races, sexes, and body weights did not differ significantly in their chemical composition. Consequently, they could be used for both training and validation data and test data for the KidneyResNet classifier.

### 3.2. Spectral Properties of the Kidneys According to the Functional Classes

**Figure 2:**
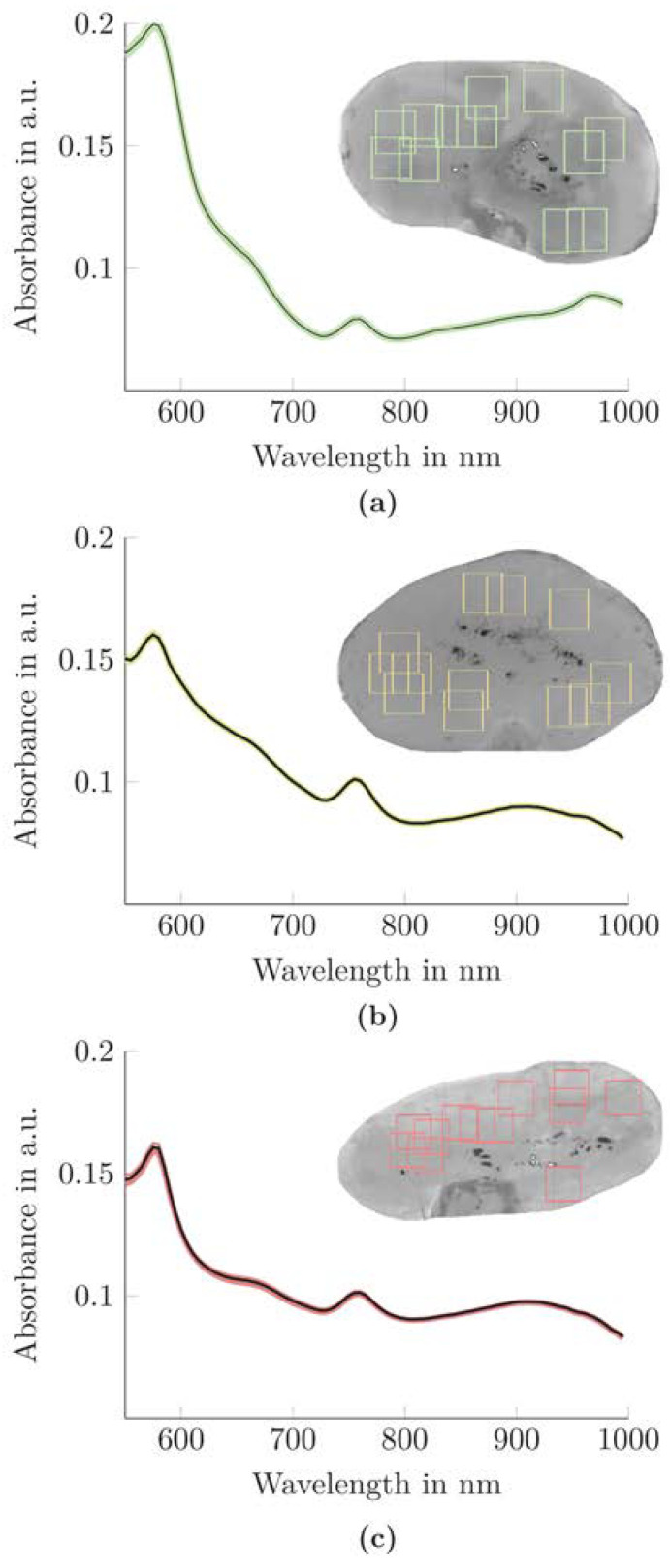
Spectral signature of kidneys with different inulin clearance behavior after 240 min NMP. The mean preprocessed absorbance spectrum of all 12 ROIs of a kidney (shown as a black solid line) and their standard deviation (shown as (**a**) green solid lines for a functional kidney, (**b**) yellow solid lines for a limited functional kidney, and (**c**) red solid lines for a nonfunctional kidney) is presented.

A qualitative analysis of the spectral curve of each functional class was performed (see Figure 2).

The kidneys’ spectra demonstrated low intra-class variation but high inter-class variation. Presumably, the low intra-class variation was mainly achieved by the selection of homogeneous ROIs. However, the inter-class differences in the spectral properties of kidneys may be beneficial for their classification with a KidneyResNet model.

### 3.3. Optimization of Classification of ROIs Using the KidneyResNet Model

In the optimization process, KidneyResNet models were trained based on absorbance and reflectance data using various training parameters and data augmentation techniques (see Section 2.8). Classification results for categorizing the individual ROIs of kidneys into three classes—functional, limited functional, and nonfunctional are shown in Table 6.

The best KidneyResNet model performance was achieved by models Nos. 3, 9, and 10 with a mean validation accuracy of 0.85, 0.84, and 0.84, respectively.

Using the KidneyResNet model configuration that performed the best in validation (see Table 6, model No. 3), data augmentation was applied. The resulting validation accuracy was consistent or worse (see Table 7). To verify this, data augmentation was also executed with the KidneyResNet model configurations of model Nos. 9 and 10 (see Table 6). The results of the KidneyResNet models could not be improved in validation by data augmentation (see Table 7).

The five optimized KidneyResNet models that yielded the best validation mean accuracy (see Table 6 models Nos. 3, 9, 10 and Table 7 models Nos. 1, 5) were selected to predict the classes of ROIs in the test data set. Here, model No. 10 from Table 6 achieved the highest accuracy, recall and precision with 62%, 58% and 58%, respectively (see Table 8). However, since these results are mean values for all ROIs of all kidneys in the test data set, they must not be projected on classification performance of a single kidney.

### 3.4. Classification of Kidneys Using the KidneyResNet Model

**Figure 3:**
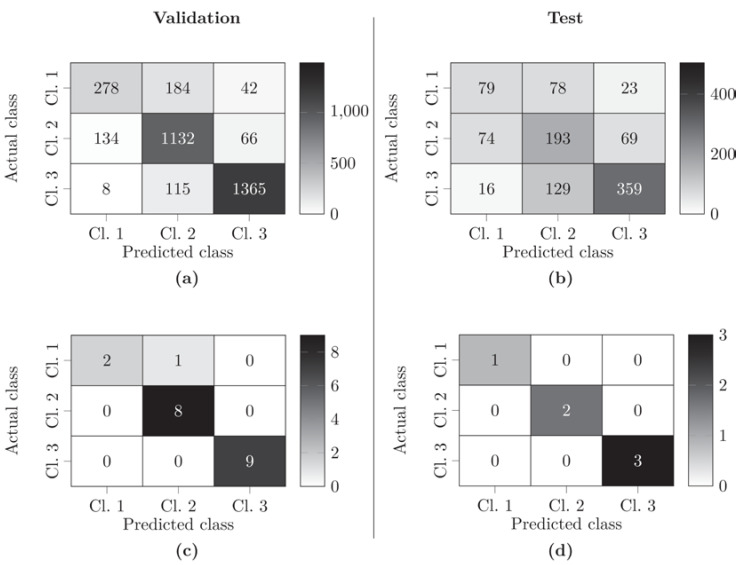
Confusion matrices for the classification of kidneys into the three classes: nonfunctional (class 1), limited functional (class 2), functional (class 3). Results of the KidneyResNet model No. 10 of Section 3.3 for validation/test after the classification of individual ROIs (**a**,**b**) and by the majority decision of all ROIs of a kidney (**c**,**d**).

To demonstrate the potential of KidneyResNet classification of HSI data in the context of NMP, the results of model No. 10 (see Table 6) were evaluated using confusion matrices (see Figure 3). The results of the validation and the test for the classification of each ROI were compared (see Figure 3a,b). As a final classification step, the classification results of all ROIs of the respective kidney were used for a majority decision to predict the functional performance of each single kidney (see Figure 3c,d).

Considering each individual ROI, the KidneyResNet allowed a reliable classification of functional (class 3) and limited-functional kidneys (class 2) (see Figure 3a,b). In the case of nonfunctional kidneys (class 1), the KidneyResNet did not allow a unanimous assignment. By applying majority decision, all but one nonfunctional kidney from the validation data set could be correctly classified according to their function (see Figure 3c,d). As a result, a KidneyResNet model accuracy of 96% for the validation data set and 100% for the test data set was achieved.

In general, the KidneyResNet model assigned the correct class to kidneys with a high degree of reliability, as calculated from the proportion of ROIs correctly classified for a kidney (see Table 9). In 42% of organs (11/26), prediction of function was achieved with a classification reliability of >90%. Of these, 72% (8/11) are functional kidneys and the remaining kidneys (3/11) were organs with limited function. Exceptions to the unambiguous classification included a nonfunctional kidney and a kidney with limited function, which had a classification reliability of <50%. Consequently, their results were based on a tendency rather than a primary clear assignment. Only one outlier existed, as the KidneyResNet model could not correctly predict the class of nonfunctional kidney No. 21 from the training and validation data set.

## 4. Discussion

The present work is the first to provide an objective classification of normothermic perfused kidneys based on their ex vivo functional status. Here, HSI in the wavelength range of 550 nm to 995 nm was used to identify function-related kidney tissue variations. To prepare HSI data as input to the machine learning algorithm, automated ROI selection was an essential preprocessing step. Different model variations were tested based on the KidneyResNet architecture and various optimization methods were applied to classify kidneys according to their inulin clearance behavior. The best model allowed a classification of the renal status into functional, limited functional, and nonfunctional. KidneyResNet in combination with HSI input appears to learn relevant spectral properties to evaluate ex vivo kidney function. Thus, this technique builds the fundament for the development of new diagnostic tools for non-invasive organ assessment.

### 4.1. Organ Classification Methods

Research in the field of organ assessment in the period between ex- and implantation is still in its early stages. Only a few studies used multivariate classifiers to predict organ injury based on specific markers.

Rat livers during a six-hour NMP were evaluated by considering the perfusion markers glucose, lactate, and urea using the partial least squares (PLS) discriminant analysis. The organs were classified with a specificity of 90% into freshly explanted organs and ischemically pre-damaged organs (WIT = 60 min to 90 min). In addition, a retrospective analysis of liver transplantability was performed using multiway principal component analysis (PCA). Perfusate markers allowed classification into three groups—freshly harvested organs and organs with 60 min and 90 min WIT [46].

Bruinsma et al. published a study in which human livers rejected for transplantation were subnormothermically perfused. Using mass spectrometry, 159 metabolites were detected, allowing classification of the livers into two groups—organs with WIT < 30 min and WIT > 30 min—by PCA [47].

Furthermore, in investigations on hypothermic machine perfusion-treated pig livers using logistic regression, Liu et al. established a damage index based on aspartate aminotransferase activity and pH in the perfusate, which could reflect organ damage caused by IRI [48].

These studies demonstrated that objective assessment of organs in the context of extracorporeal machine perfusion is possible with appropriate machine learning algorithms. The limitation of these studies is that the authors mainly focused on realizing a classification of organs depending on the ischemia time rather than the organ function itself.

Classification methods applied for medical hyperspectral imaging are, among others, support vector machines and artificial neural networks (ANN) [49]. The CNN is an ANN, that is particularly suited for interpreting hyperspectral data sets by selectively abstracting fundamental features and enabling rapid processing of large data sets [35]. In addition, modeling can be performed with both spectral and spatial features [35].

The combination of CNN and HSI has been successfully applied to assist in the diagnosis of various tissue types. Examples of histologic tissue examination include classification or segmentation of head and neck cancer [39,50], breast cancer [37,51], gastric cancer [38], oral cancer [52], esophagus [53], hepatocellular carcinoma [54], glioblastoma tumor cells [40], and blood cells [55]. Its use in neurosurgery has been investigated for in vivo real-time segmentation of the tumor margin [40]. Furthermore, research has been conducted on the classification of pathogenic bacteria [56] and corneal epithelial injury [57].

In contrast, this work is the first to objectively classify the functional state of ex vivo normothermically perfused kidneys based on measuring tissue-specific optical properties with HSI and the use of KidneyResNet. No similar applications are known in the literature.

### 4.2. ROI Selection for Preprocessing of HSI Data

Depending on the data basis and application, different methods were used in the literature to extract ROIs and different sized ROIs were chosen for data analysis.

ROIs can be determined by visual inspection confirmed by a physician [38,52,54] or by appropriate labeling of acquired images by a surgeon using a semi-automated tool based on the spectral angle mapper algorithm [40]. Masks are also used to allow ROI selection by avoiding specular reflections [39,50]. Furthermore, programs were used to select ROI (CytoSpec, ENVI) [56,58].

The spatial size of the ROI used as input to the CNN ranged from 11 px × 11 px [40,53,55] to 250 px × 250 px [52] but is mostly < (50 px × 50 px) [37,39,50,51]. The number of ROIs used varied considerably. For the ROI selection, either several ROIs [38,52] or all pixels of the measured biological object, which then represent the respective center of the ROIs [39,50,53], were selected.

In this study, ROIs of 50 px × 50 px were defined. It was possible to select up to four ROIs in the specific kidney regions with the selected ROI size. This approach allowed the objective selection of 12 ROIs from each hyperspectral image at each measurement time point, resulting in 168 ROIs of each kidney.

Automated ROI selection was implemented in the present work to develop an objective and standardized method for determining ROIs (see Figure 1). Since the computational time and effort required to develop CNN models increase with the amount of data, the hyperspectral data cubes were limited to information-containing regions. The strategy here was to first segment a background with no tissue properties and then determine only ROIs with unique homogeneous characteristics. This homogenous ROI selection maximizes the probability that the KidneyResNet will be trained on spectral rather than spatial changes. It also ensures that no specular reflections or outlier spectra are selected that could falsify the classification with KidneyResNet. Consequently, it was possible to determine a small intra-class variation in the spectral signature and thus extract the kidneys’ mainly relevant spectroscopic tissue properties (see Figure 2).

### 4.3. CNN Model Architecture and Optimization Methods

Compared to other studies that focus on the application of CNN models in HSI analysis in medical research, the ResNet-18 was selected as the network architecture in this work. The ResNet has proven to be very effective in providing high classification accuracy with resource optimized training. In 2015, this network won the prestigious ImageNet competition with an error rate of 3.57% [43]. In addition, in a study by Zhong et al. on a hyperspectral data set, higher classification accuracy was demonstrated with ResNet compared to other CNNs [59]. For this reason, we applied the ResNet-18 and did not perform any optimizations to the network architecture itself. Other research groups focused also on the number and size of convolutional kernels [38,39], the number of neurons in fully connected layer [38], and the number of convolutional layers [38,39].

In the literature, the application of different training parameters to optimize CNN models in HSI analysis was described. The following methods were mainly implemented: dropout [37,39,50,54,57], learning rate optimization [37,38,40,51,53,55,57], weight decay [51,55,57], and momentum factor [38,51]. The three most commonly used training parameters were investigated in this work for their suitability to improve the KidneyResNet model performance (see Table 2). As a result, it was found that a suitable KidneyResNet model with a dropout rate of 50% and a learning rate decay of 0.11 provided adequate classification results (see Table 6 and Table 9, model No. 10).

In addition, we investigated whether the classification result could be further improved by increasing the size of the data set using data augmentation. Commonly used data augmentation methods are rotation [37,40,51,57], vertical/horizontal flipping [37,40,51,57], and presence of Gaussian noise [53]. They are often used simultaneously and without systematic studies towards the influence of single data augmentation methods on the outcome of CNN models [37,40,51,57]. In this work, three data augmentation methods—rotation, random pixel occlusion, and presence of Gaussian noise—were investigated for their potential to optimize the KidneyResNet model (see Table 3). However, no general improvement was achieved implementing these methods (see Table 7).

### 4.4. Analysis of Exclusively Tissue-Specific Data Enables Functional Evaluation of Kidneys

The KidneyResNet classifier achieved excellent predictive results in assigning kidneys into three functional classes (see Figure 3, Table 9). The KidneyResNet correctly predicted the function of 19 of the 20 kidneys during leave-one-out cross-validation and 6 of the 6 kidneys in the hold-out test data. Consequently, for the first time the kidneys could be divided into three functional classes, and nonfunctional kidneys can be distinguished from kidneys with limited function and functional kidneys. Initial results of measured perfusion characteristics (renal blood flow, mean arterial pressure, intrarenal resistance, total urine output) showed no significant differences between limited functional and nonfunctional kidneys [18].

Due to the small data set that was available for optimization and testing of the model, these results are only proof of concept. It is very promising, that a model trained on samples from only 20 kidneys can classify validation and test data with a high reliability. However, the ranking of the kidneys by classification reliability in Table 9 shows the test data to be at the bottom half of the table. This is most likely due to the phenomenon of overfitting, which is common in machine learning and can be amended by regularization techniques. As we already tested regularization techniques (dropout rate, weight decay) without further improvements, we conclude that the most effective enhancement of our model would come from more input data for training, i.e., more HSI data from more kidneys during NMP. Also, more test data would lead to a higher statistical certainty of the resulting metrics.

Overall, the results obtained in this work suggest that an appropriate KidneyResNet model trained on HSI data can predict renal quality during NMP. Additional measurements of various markers from blood and urine were not required to ensure this evaluation. Research groups that have focused on assessing organs in NMP postulated that only a combination of different markers could provide an adequate determination of the renal status [12,13,14,15]. These research groups had previously attempted functional assessment based on hemodynamic and/or blood and urine markers. According to the current state of research, the statement that a combination of the markers above is necessary for renal quality evaluation cannot be refuted. Nevertheless, in this work, a novel evaluation strategy based solely on VIS/NIR spectroscopic tissue properties was developed and achieved excellent classification results.

In further research, the classification results obtained in this work have to be confirmed using a larger data set. This would allow the KidneyResNet to better generalize from the training data. Due to the small amount of data in the present work that is resulting in a limited number of training data (20 kidneys, 3360 ROIs) and test data (6 kidneys, 1008 ROIs), the classification quality of the models may be strongly affected by outliers. Thus, selecting the training and validation data set and test data set decisively determines the classification result. In addition, the large variability in the validation results and the lower reliability on test data shows that more data is needed to train a steady KidneyResNet model.

Furthermore, clinical follow-up studies should be performed in cases where NMP is applied before kidney transplantation. Thus, inulin clearance, the reference for the ex vivo renal function used in this work, could be compared with the postoperative in vivo function. If the inulin clearance does not prove to be the gold standard for ex vivo functional assessment, the KidneyResNet could also easily be trained on other markers (e.g., hemodynamic and/or blood and urine markers). Thus, the presented KidneyResNet model is highly adaptable depending on the reference markers used for the assessment of the renal quality.

In conclusion, the assessment strategy to determine the functional status of ex vivo kidneys presented here is based on a KidneyResNet model trained with HSI data. The spectral information of the HSI data forms the basis for classification. The here established algorithms integrate pre-processing of HSI data with a focus on automated ROI selection and training parameters that enable the optimization of the KidneyResNet model. Our optimized model shows high classification quality for differentiating kidneys according to their inulin clearance behavior.

Our approach thus demonstrates the feasibility of classifying kidneys by renal function using KidneyResNet models based on tissue-specific features. The necessary data can be determined noninvasively by HSI during NMP. Knowledge about the ex vivo renal functional status could be the first step towards the objective evaluation of ex vivo kidneys and encourage the increased use of viable organs from marginal donors in the long term.

## Figures and Tables

**Table 1:** Overview of kidneys preserved ex vivo with normothermic machine perfusion (NMP). Listed are the sex, race, warm ischemia time, cold ischemia time, glomerular filtration rate (GFR) and inulin eliminated from the blood during NMP (I_e,total_) for all kidneys of a class, kidneys in the training and validation data set, and kidneys in the test data set. Data are presented as mean ± standard deviation.

Pig and Kidney Characteristics	Class 1	Class 2	Class 3
*Overall*	*n* = 4	*n* = 10	*n* = 12
Female:Male	3:1	4:6	4:8
German Landrace:Swabian Hall	3:1	4:6	4:8
Warm ischemia time in min	122 ± 52	67 ± 30	12 ± 7
Cold ischemia time in min	404 ± 246	402 ± 201	312 ± 170
GFR in mL/min/100 g	1.3 ± 0.6	3.0 ± 0.9	14.8 ± 10.1
I_e,total_ in %	45 ± 1	72 ± 9	97 ± 3
*Training and validation*	*n* = 3	*n* = 8	*n* = 9
Female:Male	2:1	3:5	2:7
German Landrace:Swabian Hall	2:1	3:5	2:7
Warm ischemia time in min	114 ± 61	66 ± 34	12 ± 7
Cold ischemia time in min	341 ± 259	397 ± 233	305 ± 125
GFR in mL/min/100 g	1.5 ± 0.8	2.9 ± 0.8	16.4 ± 10.4
I_e,total_ in %	45 ± 1	72 ± 9	97 ± 3
*Test*	*n* = 1	*n* = 2	*n* = 3
Female:Male	1:0	1:1	2:1
German Landrace:Swabian Hall	1:0	1:1	2:1
Warm ischemia time in min	145	73 ± 10	13 ± 8
Cold ischemia time in min	594	420 ± 121	333 ± 309
GFR in mL/min/100 g	0.8	3.4 ± 1.4	10.0 ± 9.3
I_e,total_ in %	44	75 ± 12	98 ± 1

**Table 2:** Investigation matrix of the training parameters. For the optimization of the KidneyResNet, the influence of the data origin and the training parameters dropout rate, adaptive weights, and learning rate was studied.

Data Origin/	Code		Variant	
Training Parameter		I	II	III
Data origin	A	Absorbance	Reflectance	
Dropout rate in %	B	0	25	50
Weight decay	C	0	0.0005	
Learning rate	D	0	0.11	

**Table 3:** Investigation matrix of the data augmentation methods. For the enlargement of the input data of the KidneyResNet, the influence of the data augmentation methods rotation, induction of Gaussian noise, and random occlusion was investigated.

Data Augmentation	Code	Variant			
Method		I	II	III	IV
Rotation	A	0°	90°	180°	270°
Gaussian noise, 3σ	B	0	0.00625		
Random occlusion in %	C	0	25		

**Table 4:** Overview of pig characteristics. Listed are the race, sex, body weight, and ischemia time of the pigs whose kidneys are used for the spectra comparison.

Nr.	Race	Sex	Body Weight in kg	Warm Ischemia Time in min	Cold Ischemia Time in min
1	Swabian Hall	Male	40 ± 5	20	136
2	German Landrace	Female	40 ± 5	20	221
3	German Landrace	Female	80 ± 3	25	343
4	Swabian Hall	Male	40 ± 5	60	277
5	German Landrace	Female	40 ± 5	118	463
6	German Landrace	Female	80 ± 3	80	334

**Table 5:** Spectral comparison of kidneys. The spectra of kidneys as a function of race, sex, body weight, and ischemia time were examined using normalized cross-correlation. The assignment of the numbers can be found in the previously listed table.

Comparison	Pearson Correlation Coefficient	Comparison	Pearson Correlation Coefficient
1 vs. 2	0.991	4 vs. 5	0.997
1 vs. 3	0.998	4 vs. 6	0.995
2 vs. 3	0.993	5 vs. 6	0.998

**Table 6:** Classification results on the validation data set for the assignment of kidneys into three functional classes depending on different training parameter combinations. The explanations for parameters A−D and I−III are given in Section 2.8.

Model No.	Parameter				Median Early Stopping Epoch	Validation Mean Accuracy
	A	B	C	D		
1	I	I	I	I	9	0.80
2	I	I	I	II	2	0.72
3	I	I	II	I	9	0.85
4	I	I	II	II	5	0.77
5	I	II	I	I	13	0.82
6	I	II	I	II	4	0.69
7	I	II	II	I	12	0.81
8	I	II	II	II	3	0.79
9	I	III	I	I	11	0.84
10	I	III	I	II	7	0.84
11	I	III	II	I	6	0.75
12	I	III	II	II	6	0.78
13	II	I	I	I	8	0.83
14	II	I	I	II	5	0.69
15	II	I	II	I	15	0.82
16	II	I	II	II	4	0.72
17	II	II	I	I	9	0.79
18	II	II	I	II	4	0.71
19	II	II	II	I	12	0.81
20	II	II	II	II	3	0.74
21	II	III	I	I	10	0.79
22	II	III	I	II	3	0.69
23	II	III	II	I	12	0.81
24	II	III	II	II	4	0.73

**Table 7:** Classification results on the validation data set for the assignment of kidneys into three functional classes depending on the data augmentation methods. The explanations for parameters A−C and I−IV are given Section 2.8.

Model No.	Parameter			Median Early Stopping Epoch	Validation Mean Accuracy
	A	B	C		
*Input KidneyResNet model configuration: model No. 3, Table 6*					
1	I–IV	I	I	7	0.85
2	I	II	I	5	0.73
3	I	I	II	6	0.83
*Input KidneyResNet model configuration: model No. 9, Table 6*					
4	I–IV	I	I	10	0.81
5	I	II	I	10	0.84
6	I	I	II	10	0.79
*Input KidneyResNet model configuration: model No. 10, Table 6*					
7	I–IV	I	I	3	0.65
8	I	II	I	2	0.78
9	I	I	II	4	0.67

**Table 8:** Classification results on the test data set for the assignment of kidneys into three functional classes.

Model No.	Test		
	Accuracy	Recall	Precision
*Table 6*			
3	0.28	0.25	0.29
9	0.41	0.32	0.32
10	0.62	0.58	0.58
*Table 7*			
1	0.55	0.49	0.56
5	0.59	0.48	0.49

**Table 9:** Overview of the 3-class division of kidneys with a KidneyResNet. The kidneys of the test data set are marked with *. Note that all kidneys without * were part of the training process. The color coding corresponds to the functional status of the kidneys: red = nonfunctional kidneys (class 1), yellow = limited functional kidneys (class 2), green = functional kidneys (class 3). The actual class refers to the functional classes of the kidneys as determined by the clinical gold standard, which is compared to the predicted class resulting from the KidneyResNet analysis. In addition, the classification reliability is presented, which corresponds to the proportion of correctly classified ROIs of a kidney. A diagonal line represents kidneys that could not be assigned to the correct functional class.

Kidney No.	Actual Class	Predicted Class	Classification Reliability	
1	3	3	99%	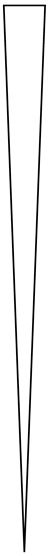
2	3	3	99%	
3	3	3	98%	
4	3	3	98%	
5	2	2	98%	
6	2	2	97%	
7	3	3	96%	
8	2	2	94%	
9	3	3	93%	
10	3	3	92%	
11 *	3	3	91%	
12	2	2	88%	
13	2	2	86%	
14	3	3	84%	
15	2	2	81%	
16	2	2	80%	
17	1	1	71%	
18 *	2	2	70%	
19 *	3	3	69%	
20	3	3	66%	
21	1	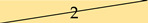	64%	
22	1	1	61%	
23	2	2	57%	
24 *	3	3	54%	
25 *	1	1	44%	
26 *	2	2	43%	

## Data Availability

All data will be made available upon reasonable request to the corresponding author.

## Supplementary Materials

The following supporting information can be downloaded at: https://www.mdpi.com/article/10.3390/biomedicines10020397/s1, Table S1: KidneyResNet model architecture.
