# Supplementary material for: Hyperspectral Imaging during Normothermic Machine Perfusion—A Functional Classification of Ex Vivo Kidneys Based on Convolutional Neural Networks
Source: Biomedicines. 2022 Feb 7;10(2):397. doi: 10.3390/biomedicines10020397 (PMC8962340; doi:10.3390/biomedicines10020397)
Supplement: Supplementary file 1 [file biomedicines-10-00397-s001.zip › biomedicines-1551557-supplementary.pdf]

Supplementary Material

CNN Model Architecture

Table S1. KidneyResNet model architecture.

Layer Name	Output Size	Comment
Convolution 2D-Input Layer	64 × 25 × 25	[Convolutional layer 1] Kernel size 3, stride 2, variable drop-out-rate, activation ReLu
Residual block 1	64 × 25 × 25<br>64 × 25 × 25	[Convolutional layer 2] Kernel size 3, stride 1, variable drop-out-rate, activation ReLu<br>[Convolutional layer 3] Kernel size 3, stride 1, variable drop-out-rate, activation ReLu
Pooling layer 1	64 × 25 × 25	2 × 2 Max pooling
Residual block 2	128 × 25 × 25<br>128 × 25 × 25<br>128 × 25 × 25	[Convolutional layer 4] Kernel size 3, stride 1, variable drop-out-rate, activation ReLu<br>[Convolutional layer 5] Kernel size 3, stride 1<br>[Convolutional layer 6] Identity convolution: kernel size 1, stride 1, variable dropout-rate, activation ReLu
Pooling layer 2	128 × 25 × 25	2 × 2 Max pooling
Residual block 3	256 × 25 × 25<br>256 × 25 × 25<br>256 × 25 × 25	[Convolutional layer 7] Kernel size 3, stride 1, variable drop-out-rate, activation ReLu<br>[Convolutional layer 8] Kernel size 3, stride 1<br>[Convolutional layer 9] Identity convolution: kernel size 1, stride 1, variable dropout-rate, activation ReLu
Pooling layer 3	256 × 25 × 25	2 × 2 Max pooling
Residual block 4	512 × 25 × 25<br>512 × 25 × 25<br>512 × 25 × 25	[Convolutional layer 10] Kernel size 3, stride 1, variable drop-out-rate, activation ReLu<br>[Convolutional layer 11] Kernel size 3, stride 1<br>[Convolutional layer 12] Identity convolution: kernel size 1, stride 1, variable dropout-rate, activation ReLu
Pooling layer 4	512 × 25 × 25	1 × 1 Average pooling
Flatten	320,000 × 1	
Fully connected layer	3	Activation Softmax
